# Prognostic predictive value of intracranial pressure and cerebral oxygen metabolism monitoring in patients with spontaneous intracerebral hemorrhage

**DOI:** 10.1007/s13760-022-02037-5

**Published:** 2022-08-27

**Authors:** Zhen Sun, Jing Liu, Shengpu Dong, Xianglong Duan, Fei Xue, Xingyu Miao

**Affiliations:** 1 Shaanxi Provincial People’s Hospital, Xi’an, 710086 Shaanxi China; 2 Zhujiang Hospital of Southern Medical University, Guangzhou, 510280 Guangdong China

**Keywords:** Spontaneous intracerebral hemorrhage, Intracranial pressure, Cerebral oxygen metabolic indexes, Combined prognosis prediction

## Abstract

**Objective:**

Our study aimed to investigate the predictive value of intracranial pressure (ICP) and cerebral oxygen metabolism monitoring in the postoperative prognosis of patients with spontaneous intracerebral hemorrhage (SICH).

**Methods:**

The clinical data of 55 patients with SICH treated by neurosurgery were analyzed retrospectively. These patients were divided into two groups based on postoperative Glasgow Outcome Scale (GOS) scores, i.e., the poor prognosis group (GOS I-III) and the good prognosis group (GOS IV and V). Next, the ICP and cerebral oxygen metabolism indexes, such as brain temperature (BT), cerebral perfusion pressure (CPP), internal jugular venous oxygen saturation (SjvO_2_), and arterial partial pressure of carbon dioxide (PaCO_2_), were recorded after the operation. Further, the prognostic differences between the two groups were compared, and the predictive values were evaluated using the receiver operating characteristic curve (ROC) and area under the curve (AUC).

**Results:**

The results showed that the average ICP and BT in the good prognosis group were lower than those in the poor prognosis group. However, the CPP and SjvO_2_ in the good prognosis group were higher than those in the poor prognosis group. Moreover, the incidence of low PaCO_2_ in the poor prognosis group was higher than that in the good prognosis group.

**Conclusions:**

Our results demonstrated that the average ICP, BT, CPP, SjvO_2_, and arterial PaCO_2_ may reflect the changes in brain function and cerebral blood flow, which are significantly correlated with the prognosis of patients. Further, our findings indicated that the combined postoperative ICP levels with cerebral oxygen metabolism indexes could guide clinical treatments and predict prognosis.

## Introduction

Spontaneous intracerebral hemorrhage (SICH) is a cerebral hemorrhage caused by arterial rupture and is characterized by high morbidity, mortality and disability rate. The incidence of SICH in patients in Europe and the US is 9–28% whereas in China, it is 19–48% [1, 2]. According to a report, the mortality rate of patients with SICH within 30 days is approximately 45% [3]. Although the mortality rate has been reduced owing to surgical intervention, the incidence of neurological function disorders in patients with SICH is still relatively high. Thus, SICH is considered to be one of the most challenging public health problems worldwide [4, 5]. A high intracranial pressure (ICP) was shown to be an independent risk factor for poor prognosis after SICH, and patients with long-term ICP > 22 mmHg require medical intervention to reduce ICP [6, 7]. However, many cerebral oxygen metabolic indexes vary, which eventually leads to ICP instability. In this study, we retrospectively analyzed a series of intracranial physiological and pathological indexes (ICP, brain temperature (BT), cerebral perfusion pressure (CPP), internal jugular venous oxygen saturation (SjvO_2_), and arterial partial pressure of carbon dioxide (PaCO_2_)) in patients with SICH. Further, the study explored the role of ICP in combination with cerebral oxygen metabolism indexes to predict the prognosis of patients with SICH.

## Data and methods

### Patient information and grouping

The clinical data of 55 patients with SICH, including 27 males and 28 females who underwent surgery at the Department of Neurosurgery of Shaanxi Provincial people’s Hospital from June 2020 to December 2021, were analyzed retrospectively. According to the guidelines [8], all patients were administered the basic treatment of hemostasis and intravenous antihypertensive therapy. Blood pressure was controlled under 140 mmHg to prevent hematoma expansion and nervous system deterioration, to promote functional recovery. The Glasgow Coma Scale (GCS) score of the patients was between 4 and 9. According to the Glasgow Outcome Scale (GOS) scores, patients were divided into two groups at 3 month postoperatively: the poor prognosis group (GOS I–III) and the good prognosis group (GOS IV–V). The study methods complied with the principles of the Helsinki Declaration. With the approval of the hospital ethics committee, family members of each patient provided signed informed consent for operation and placement of the ICP probe. The inclusion criteria of the study were as follows: (1) Brain CT scan indicating symptoms of intracranial hemorrhage, (2) patients with emergency surgical indications, including evacuation of intracranial hematoma, decompression of bone flap or minimally invasive neuroendoscopic surgery, and intraventricular ICP probe with brain temperature monitoring function placed after the operation, (3) central venous catheters placed in patients after the operation, and (4) intracranial pressure, cerebral oxygen metabolism, and arterial blood gas were monitored for 3 consecutive days after the operation. However, the exclusion criteria were as follows: (1) previous surgical history, (2) patients with multiple organ failures or other serious underlying diseases who cannot tolerate surgical treatment, (3) patients long-term anticoagulant or antiplatelet drug treatment, (4) recurrent stroke within 3 months.

All patients were transferred to the ICU after the operation and the head CT scan was reexamined within 12 h postoperatively. Moreover, the ICP, BT, and mean  arterial pressure (MAP) were collected per hour and SjvO_2_ and arterial PaCO_2_ were monitored every 8 h. Further, the average ICP, BT, CPP (CPP = MAP-ICP), SjvO_2_, and arterial PaCO_2_ were calculated.

No postoperative recurrent cerebral hemorrhage was observed in any patient. However, one patient died in the poor prognosis group, whereas no death or loss of mobility state was observed in the good prognosis group.

Some specific treatments were administered as follows:

1. When the postoperative ICP was higher than 22 mmHg and lasted for more than 15 min, dehydration drugs were administered intravenously, and analgesic and sedative drugs were also administered intravenously.
2. When the postoperative ICP was higher than 30 mmHg, intermittent and rapid administration of multiple dehydration drugs, cerebrospinal fluid drainage, analgesic and sedative therapy, and hyperventilation therapy was implemented.
3. When the postoperative ICP was higher than 45 mmHg, the head CT scan was reexamined and reoperation was considered according to the situation.
4. "Postoperative brain temperature management": the BT was controlled at 37℃. However, when the BT was above 37℃, an ice cap or ice blanket was used.

### Data extraction

The ICP probe with BT monitor implanted into the ventricle of patients after the operation was removed after 3 days. The ICP and BT data stored on the intracranial pressure monitor were collected hourly, and the average value was calculated. The CPP obtained was the difference between the MAP and ICP. Besides, central venous catheterization was performed in patients, and the SjvO_2_ value was obtained every 8 h. Meanwhile, arterial blood gas analysis was performed to obtain PaCO_2_.

### Statistical analysis

SPSS 22.0 software was used for statistical analysis. A Student’s *t*-test was used to compare the measurement data following normal distribution, and the data were expressed in terms of mean ± standard deviation. The Mann_Whitney *U* test was used to analyze the non-normal distribution of measurement data between groups, and the data were expressed using median (M) and quartile (IQR). Moreover, the classified variables were expressed by the number of cases and percentage or constituent ratio. The chi-square and Fisher’s exact probability tests were used for comparison. A *P*_value < 0.05 was considered statistically significant. The MedCalc software (version 19.0.2) was used to prepare the subject receiver operating characteristic curve (ROC), and the area under the ROC curve (AUC) was calculated to evaluate the significance of each index in predicting the prognosis of patients.

## Results

### Comparison of baseline and cerebral oxygen metabolism indexes between the two groups

No significant difference in age, sex, and GCS score was observed between the good prognosis group (*n* = 31) and the poor prognosis group (*n* = 24). However, the average ICP and BT in the good prognosis group (13.42 ± 3.62 *vs* 27.21 ± 7.87, *P* < 0.001) were significantly lower than those in the poor prognosis group (36.98 ± 0.54 *vs* 38.36 ± 0.65, *P* < 0.001). The CPP and SjvO_2_ of the good prognosis group (69.32 ± 4.94 *vs* 56.67 ± 10.09, *P* < 0.001) were higher than those of the poor prognosis group (65.58 ± 7.13 *vs* 48.92 ± 10.87, *P* < 0.001), with a statistically significant difference. The incidence of low PaCO_2_ in the poor prognosis group was significantly higher than that in the good prognosis group (*χ*^2^ = 16.897, *P* < 0.001), as shown in Table 1.

**Table 1:** Comparison of baseline and cerebral oxygen metabolism indexes between the two groups

Influencing factors	Age (years)		Gender		GCS score on admission M(IQR)	Mean ICP (mmHg)	
	≥ 60	< 60	Male	Female			
Good prognosis group (31 cases)	16	15	17	14	7(1)	13.42 ± 3.62	
Poor prognosis group (24 cases)	14	10	10	14	7(1.75)	27.21 ± 7.87	
Result	*χ*^2^ = 0.246		*χ*^2^ = 0.939		*U* = 310	*t* = 7.953	
*P*	0.412		0.243		0.264	< 0.001	

Influencing factors	BT(℃)		CPP(mmHg)		SjvO2(%)	Low PaCO2	
						Yes	No
Good prognosis group(31 cases)	36.98 ± 0.54		69.32 ± 4.94		65.58 ± 7.13	4	27
Poor prognosis group (24 cases)	38.36 ± 0.65		56.67 ± 10.09		48.92 ± 10.87	16	8
Result	*t* = 8.634		*t* = 5.644		*t* = 6.851	*χ*^2^ = 16.897	
*P*	< 0.001		< 0.001		< 0.001	< 0.001

**Table 2:** The role of different monitor indexes in predicting the poor prognosis of patients with SICH

Influencing factors	AUC	Sensitivity (%)	Specific degrees (%)	95% CI	*P*
Mean ICP	0.933	100	87.50	0.832 ~ 0.983	< 0.001
BT	0.939	87.10	91.67	0.840 ~ 0.986	< 0.001
CPP	0.848	100	83.33	0.726 ~ 0.931	< 0.001
SjvO2	0.890	100	87.5	0.776 ~ 0.958	< 0.001
Low PaCO2	0.769	87.10	66.67	0.636 ~ 0.872	< 0.001

### Comparison of different monitoring indexes for predicting poor prognosis of patients

**Fig. 1:**
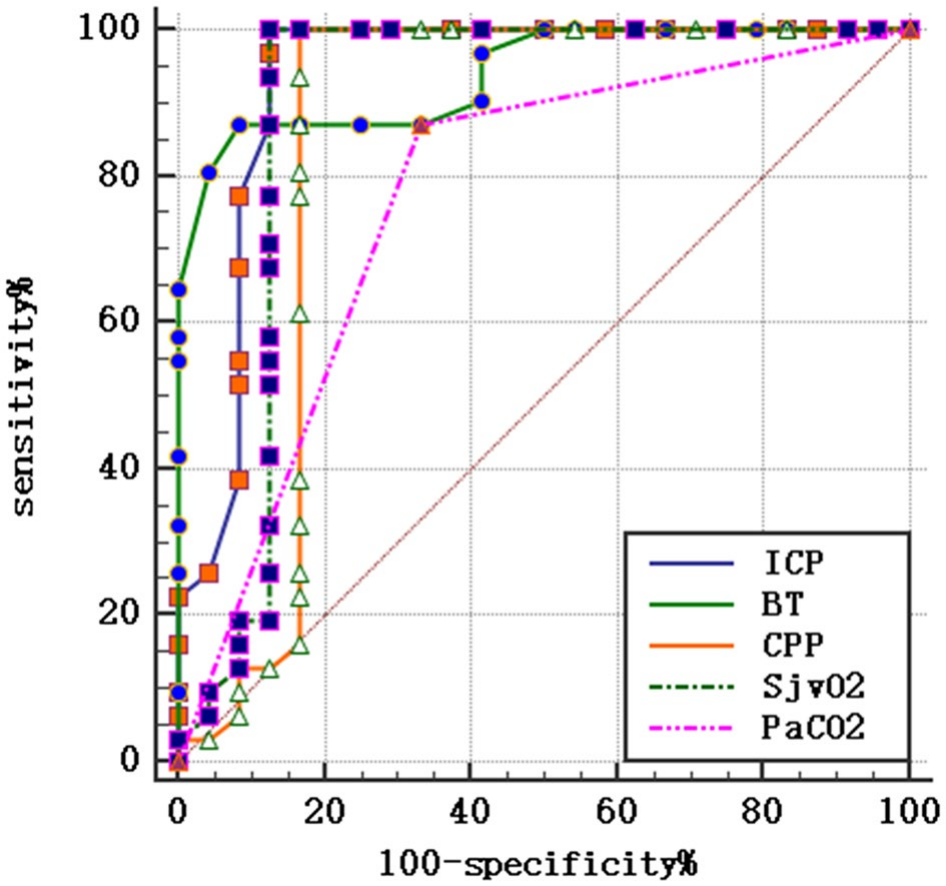
ROC of different monitor indexes in predicting poor prognosis of patients with SICH

To analyze the significance of different indexes on the poor prognosis of patients with SICH after operation, the average values of indexes were compared which were as follows: ICP (AUC = 0.933, 95% CI 0.832–0.983, *P* < 0.001), BT (AUC = 0.939, 95% CI 0.840–0.986, *P* < 0.001), CPP (AUC = 0.848, 95% CI 0.726–0.931, *P* < 0.001), SjvO_2_ (AUC = 0.890, 95% CI 0.776–0.958, *P* < 0.001) and low PaCO_2_ (AUC = 0.769, 95% CI 0.636–0.872, *P* < 0.001). The results revealed that the combination of ICP levels with cerebral oxygen metabolic indexes is of significant importance for predicting poor prognosis in patients with SICH, as shown in Table 2. Fig. 1.

## Discussion

SICH can cause a series of pathophysiological changes that are usually characterized by the rapid expansion of hematoma in the brain parenchyma, which extends to the ventricular system and subarachnoid space or dural space and leads to a high risk of recurrent cerebral hemorrhage, serious vascular events, epilepsy, dementia, and other neurological complications [9]. Acute hemorrhage is known to damage brain tissue and nerve system, leading to brain dysfunction. However, a large-sized hematoma oppresses the surrounding brain tissue and increases the pressure gradient between the hematoma area and the surrounding tissue, forcing the brain tissue to shift. The obstruction of peripheral vascular circulation, brain ischemia, and hypoxia result in brain edema, leading to a sharp increase in ICP, and eventually to brain hernia. Moreover, the acute and chronic toxic effects of hematoma decomposition lead to brain edema, degeneration, and necrosis, which further increases the ICP levels and aggravates neurological dysfunction. Therefore, it is necessary to remove intracerebral hematoma as early as possible to relieve compression and reduce the ICP levels and cytotoxicity of blood components, which preserves nerve function to the maximum extent and creates favorable conditions for the recovery of brain function [10]. At present, surgical treatments, including craniotomy, minimally invasive surgery, and bone flap decompression, are the main therapeutic strategies for SICH. Irrespective of the kind of surgical treatments, patients with SICH still have postoperative complications resulting in increased ICP levels, disturbance of cerebral oxygen metabolism and insufficient cerebral perfusion which affects the postoperative neurological functions and the quality of patients’ life. Therefore, during treatment after SICH, along with the fluctuation of ICP, the hemodynamic indexes reflecting the physiological and pathological of cerebrovascular should also be monitored. ICP, BT, CPP, SjvO_2_, and PaCO_2_ were found to be of great significance in predicting the prognosis of traumatic brain injury (TBI) [11]. Our study evaluated the significance of these parameters to predict the prognosis of patients with SICH.

Secondary brain injury caused by increased ICP levels is one of the main causes of poor prognosis in patients with SICH. High ICP levels increase the inhomogeneity of capillary permeability, which causes disorders of cerebral microcirculation, blood shunt, and suboptimal transport of energy substrates. The average ICP levels can indicate the occurrence and development of secondary damage and predict the optimal time point for intervention [12], which is very important for determining the time of surgery, standardizing the dose of dehydration drugs, and improving prognosis. For the prevention and treatment of secondary injuries in patients with SICH, the best CPP should be maintained while controlling the BP. The control of ICP levels alone without CPP may lead to insufficient perfusion, which increases the risk of cerebral ischemia, thus affecting the prognosis [13]. Besides, physiological cerebrovascular dysfunction is also an important factor in the pathogenesis of SICH. CPP levels indirectly reflect cerebral blood flow (CBF) and cerebral oxygen supply as well as the response of cerebrovascular autoregulation to blood pressure fluctuation, which can predict the deterioration of intracranial disease and suggest the outcome of the disease. Neurological dysfunction and secondary brain injury are attributed to the decrease in CBF and microcirculation perfusion, which further worsen brain edema and form a vicious circle. Therefore, clinical treatment under the guidance of CPP has been widely promoted [14]. At present, the CPP threshold has been determined to be 60–70 mmHg under TBI therapy, whereas the levels are not unified under the SICH treatment. The results of our study showed that the average CPP of the good prognosis group was significantly higher than that of the poor prognosis group, whereas the average ICP of the good prognosis group was lower than that of the poor prognosis group. This suggests that sufficient CPP should be ensured and specific CBF should be maintained to reduce secondary brain injury in patients with SICH [15].

BT is closely related to brain metabolism and the intracranial environment. An increase in BT may reflect the fluctuation in brain metabolism, hyperemia, or local inflammation. The increase in temperature leads to an increase in cerebral blood perfusion. This high cerebral blood volume may increase ICP [16]. Thus, an increase in BT results in the following aspects: (1) elevated levels of excitatory amino acids (such as glutamic acid and dopamine), free radicals, lactic acid, and pyruvate, (2) increased ischemic depolarization, (3) destruction of blood_brain barrier, (4) inhibition of protein kinase and activation of some matrix metalloproteinases, and (5) decreased cytoskeleton stability. Therefore, CBF and metabolism are thought to be the main factors in brain temperature regulation. During cerebral hypoperfusion or ischemia, a reduction in CBF may increase the brain temperature [17]. The increase in BT is related to diffused depolarization, which is similar to the active process of high metabolism. Neurons remain in a state of depolarization for a long time, and the cytotoxicity increases in severity, which eventually leads to necrosis or apoptosis and causes irreversible damage to neurons [18]. Our study revealed that the average BT of the good prognosis group was significantly lower than that of the poor prognosis group. Moreover, the ROC curve indicated that the prediction of prognosis by BT is highly significant.

The artificial hyperventilation is used after craniocerebral surgery in addition to dehydration and diuresis to reduce ICP through cerebral vasoconstriction and cerebral perfusion. However, inappropriate hyperventilation can lead to a significant decrease in CBF and an imbalance between cerebral oxygen supply and demand, resulting in neurological complications and increased mortality and disability rates [19]. Therefore, it is essential to monitor CBF and oxygen metabolism after craniocerebral surgery. SjvO_2_ monitoring is a technique for estimating the overall balance between brain oxygen supply and metabolic needs. Nearly 80% of brain tissue reflux venous blood flows rapidly to the jugular bulb through the venous sinus with little extracerebral venous blood mixing [20]. Normally, the SjvO_2_ value is between 55 ~ 75%. A value less than 55% indicates that the patient has insufficient CBF owing to various reasons (hyperventilation, decreased CPP, and vasospasm) or increased metabolic oxygen demand (CMRO_2_). A value greater than 75% signifies hyperemia, reduced metabolic requirements of the brain (cell death or mitochondrial dysfunction), and microvascular shunts caused by disturbance of oxygen extraction and diffusion in the damaged brain tissue. These secondary lesions lead to poor neurological prognosis. Thus, SjvO_2_ monitoring helps determine the balance between CBF and CMRO_2_, monitor insufficient or excessive cerebral perfusion, and guide the treatment and prevention of secondary brain injury [21].

CBF is primarily regulated by arterial PaCO_2_. Hypocapnia (PaCO_2_ < 35 mmHg) may be a common and neglected cause of brain hypoxia after SICH, which is related to poor neurological prognosis and delayed cerebral ischemia [22]. Hypocapnia leads to cerebrospinal fluid alkalosis which affects the nervous system such as cerebrospinal fluid alkalosis reduces CBF through cerebral arterial vasoconstriction, and reduces cerebral blood volume to a lesser extent, resulting in reduced oxygen delivery as seen in patients with brain injury. CBF was observed to decrease approximately 3% as per change in 1 mmHg PaCO2 in patients with brain injury. Secondly, moreover, hypocapnia increases brain oxygen demand by increasing neuronal excitability or inducing seizures [23]. Hypocapnia is also associated with increased CMRO2. Thus, the decrease in oxygen delivery and the increase of CMRO_2_ lead to the imbalance of blood flow-metabolism, resulting in the transition to anaerobic metabolism or leading to cerebral ischemia, especially in the brain tissue with pre-existing CBF damage. Therefore, cerebrospinal fluid alkalosis can lead to neurotoxicity, especially through the production of excitatory amino acids, such as N-methyl-d-aspartic acid, which have severe cell cytotoxicity [24]. This suggests that hyperventilation should be carried out under the monitor of cerebral oxygenation indexes (partial pressure of brain oxygen or internal jugular venous oxygen saturation) to avoid cerebral hypoxia and increase the prognosis.

Craniotomy is the main surgical method for the treatment of SICH and the hematoma can be removed thoroughly. Although some shortcomings of craniotomy should be paid attention, including severe trauma, obvious brain tissue injury, high blood loss, long operation time, severe brain edema reaction, many complications, poor prognosis and curative effect [25]. SICH commonly occurs in deep brain tissue, such as the basal ganglia and thalamus and to remove the hematoma from the deeper areas, a large layer of brain tissue must be punctured during the operation, which may lead to iatrogenic damage to healthy brain tissue. In addition, postoperative complications under such clinical situations are common like rebleeding and infection which increases mortality, leading to poor prognosis [26]. In recent years, with the development of imaging technology, neuroendoscopy is combined with microneurosurgery, neuronavigation and intraoperative ultrasound, which makes it possible to fulfill the advantages of accurate location, less trauma and good curative effect in operations. During the endoscopic surgery of patients with SICH, the endoscope can be placed into the hematoma cavity which exposes the operation field in a panoramic manner and avoids the residual hematoma and further reduce the damage to healthy brain tissue and surgical trauma. Rapid removal of hematoma can reduce nerve damage, high-definition imaging can quickly identify active bleeding points, which is beneficial to hemostasis and reduces the risk of secondary bleeding', the process is crucial for improving the prognosis and living ability of patients [27]. The advantages of neuroendoscopic surgery have been recognized increasingly by neurosurgeons in the treatment of patients with SICH. In this study, six patients treated with neuroendoscope had good postoperative indexes and better prognosis. However, the sample size studied was too small and needs further validation.

## Conclusions

The prognosis of patients with SICH is often poor and challenging. Our study assessed a variety of monitoring methods which are used to reflect different pathophysiological conditions in postoperative patients with SICH and explored their predictive values. It was suggested that the average ICP indicated intracranial space-occupying which guided dehydration and surgical treatment. Similarly, BT and CPP suggested hemodynamic changes, guided treatments and predicted prognosis. Moreover, the levels of SjvO2 and arterial PaCO2 suggested the changes of cerebral oxygen metabolism and CBF. Further, ROC curve determined that combined postopertaive ICP levels with cerebral oxygen metabolism indexes such as CPP, BT, SjvO2 and arterial PaCO2 could guide the clinical intervention and predict the prognosis of patients with SICH more effectively.
